# The unrecognized role of fidelity in effectiveness-implementation hybrid trials: simulation study and guidance for implementation researchers

**DOI:** 10.1186/s12874-023-01943-3

**Published:** 2023-05-13

**Authors:** Diana Trutschel, Catherine Blatter, Michael Simon, Daniela Holle, Sven Reuther, Thekla Brunkert

**Affiliations:** 1 Nursing Science (INS), Department of Public Health, University of Basel, Bernoullistrasse 28, Basel, CH-4056 Switzerland; 2 Department of Nursing Science, University of Applied Sciences (HS Gesundheit), Gesundheitscampus 6-8, 44801 Bochum, Germany; 3 Städtische Seniorenheime Krefeld, De-Greiff-Str. 194, 47803 Krefeld, Germany; 4 University Department of Geriatric Medicine FELIX PLATTER, Burgfelderstrasse 101, Basel, 4055 Switzerland

**Keywords:** Implementation research, Fidelity, Effectiveness- implementation hybrid design, Simulation study

## Abstract

**Background:**

Effectiveness-implementation hybrid designs are a relatively new approach to evaluate efficacious interventions in real-world settings while concurrently gathering information on the implementation. Intervention fidelity can significantly influence the effectiveness of an intervention during implementation. However little guidance exists for applied researchers conducting effectiveness-implementation hybrid trials regarding the impact of fidelity on intervention effects and power.

**Methods:**

We conducted a simulation study based on parameters from a clinical example study. For the simulation, we explored parallel and stepped-wedge cluster randomized trials (CRTs) and hypothetical patterns of fidelity increase during implementation: slow, linear, and fast. Based on fixed design parameters, i.e., the number of clusters (C = 6), time points (T = 7), and patients per cluster (n = 10) we used linear mixed models to estimate the intervention effect and calculated the power for different fidelity patterns. Further, we conducted a sensitivity analysis to compare outcomes based on different assumptions for the intracluster-correlation coefficient and the cluster size.

**Results:**

Ensuring high fidelity from the beginning is central to achieve accurate intervention effect estimates in stepped-wedge and parallel CRTs. The importance of high fidelity in the earlier stages is more emphasized in stepped-wedge designs than in parallel CRTs. In contrast, if the increase of fidelity is too slow despite relatively high starting levels, the study will likely be underpowered and the intervention effect estimates will also be biased. This effect is more accentuated in parallel CRTs, here reaching 100% fidelity within the next measurement points is crucial.

**Conclusions:**

This study discusses the importance of intervention fidelity for the study`s power and highlights different recommendations to deal with low fidelity in parallel and stepped-wedge CRTs from a design perspective. Applied researchers should consider the detrimental effect of low fidelity in their evaluation design. Overall, there are fewer options to adjust the trial design after the fact in parallel CRT as compared to stepped-wedge CRTs. Particular emphasis should be placed on the selection of contextually relevant implementation strategies.

**Supplementary Information:**

The online version contains supplementary material available at 10.1186/s12874-023-01943-3.

## Introduction

Implementation science aims to promote the implementation of scientific evidence into real-world settings by studying factors and strategies that influence the uptake, implementation, and sustainment of interventions [1]. In contrast to clinical efficacy studies, which strive to maximize internal validity, implementation research works with and in real-world conditions exploring external validity. When moving interventions from the controlled trial world to a practice setting, contextual influences play a major role and need to be considered in the study design and methods [2].

Effectiveness-implementation hybrid designs are a relatively new approach to evaluate efficacious interventions in real-world settings while concurrently gathering information on the implementation and context [3]. In comparison to pragmatic trials, which primarily focus on effectiveness outcomes, hybrid designs combine elements of effectiveness research with implementation aspects and outcomes. Type I hybrid studies focus primarily on determining the effectiveness and secondly on collecting contextual information e.g., barriers and facilitators to implementation. In type II hybrid studies measuring effectiveness and implementation outcomes are equally important, while type III hybrid studies primarily focus on testing implementation strategies [3]. One aspect that can significantly influence the effectiveness of an intervention in real-world settings is the intervention fidelity during implementation. Fidelity can be defined as “the degree to which an intervention is carried out as it was described and originally tested and/or as the developer intended” [4]. There is an ongoing debate about the trade-off between fidelity and the necessary adaptation of an intervention to fit the implementation context [5]. To avoid this issue during evaluation, it has been recommended to define essential core functions and forms of the intervention before implementation [6]. Additionally, tailored implementation strategies can increase the fit between intervention and context [5, 7].

Meanwhile, from a measurement perspective, the time span between directed implementation efforts and the eventual full implementation of new interventions remains critical, as it depicts an evolving process with high variability. Previous research has shown that participants` responsiveness, recruitment, context, comprehensive policy description, and strategies to facilitate implementation are potentially moderating factors of fidelity [8, 9]. Therefore, researchers planning to conduct a hybrid design study need to consider the high potential for variations in the implementation process. Within the paradigm of hybrid designs a range of study designs can be used for evaluation, yet parallel cluster randomized controlled trials and stepped- wedge designs are the most common designs in evaluating the effectiveness of implementation interventions [10–12].

Current approaches to sample size calculation in these study designs were developed with clinical efficacy research in focus [13, 14]. Further, they assume high internal validity and perfect implementation at the time point when the intervention is rolled out. However, as alluded before, implementing interventions in real-world settings rarely is a straightforward process and might be prone to a range of contextual influences potentially slowing down or even preventing implementation [15].

This article aims to provide guidance for applied researchers which design parameters have to be considered when planning an implementation evaluation in the context of hybrid study designs. To highlight the importance of different implementation trajectories related to intervention fidelity, we will introduce a practical example from our own research, the FallDem study [16], perform a computer simulation quantifying the effect of different fidelity patterns on the outcome of a study in stepped-wedge and parallel cluster-randomized trials (CRT) and provide a tutorial of the performed simulation in additional file 2.

## The FallDem study

**Fig. 1:**
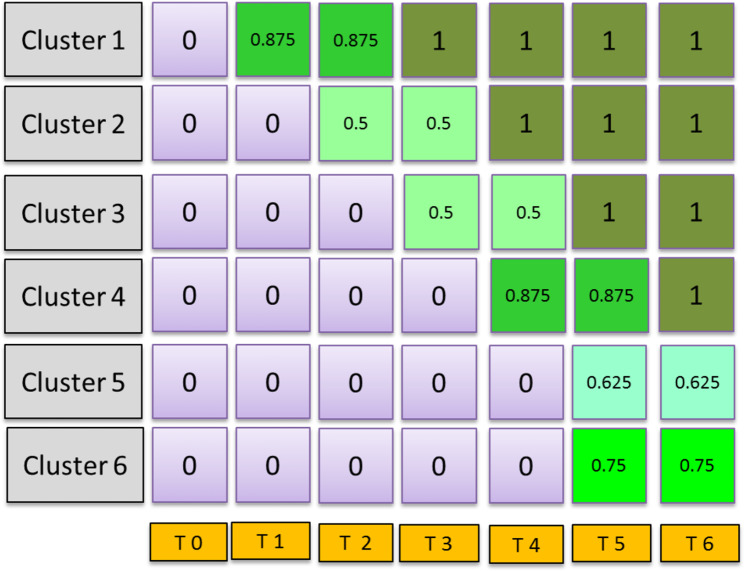
Design matrix of the FallDem study The FallDem Study is an example of a stepped-wedge CRT (with 7 time points and 6 cluster for each intervention). Fidelity was estimated from the process evaluation and provided as fractional values, which multiplied by 100 is the percentage of implementation for each time point and cluster

The overall aim of the FallDem study was to test the effectiveness of two different approaches for dementia-specific case conferences (WELCOME-IdA; WELCOME-NEO) in nursing homes (NH) [16]. The two intervention arms rely on different approaches to assess residents` challenging behaviour and its potential triggers: WELCOME-IdA included a structured assessment instrument to describe and analyse the behaviour [17], whereas in WELCOME-NEO a narrative approach was used for the analytical approach. Both intervention arms were facilitated by in-service training and on the job training during four consecutive case conferences, further implementation strategies comprised kick-off meetings with the staff, coaching of the steering group (stakeholders of the NH responsible for the implementation), trainings in moderation techniques, telephone reminders and a telephone hotline for prompt help [16]. The intervention was evaluated using a stepped-wedge CRT with seven measurement points, each three months apart. The study design was chosen with regard to the complexity related to implementing this intervention. At each of the time points, one nursing home switched from the control to the intervention phase. At the last time point, two nursing homes started with the intervention because one nursing home was included as a potential replacement for other nursing homes in the study. Overall, six nursing homes for each intervention arm (Fig. 1) with an average of 30 residents with a diagnosis of dementia (N = 404) residents [18] were included. The primary outcome was the prevalence of residents´ challenging behaviour based on the nursing home version of the neuropsychiatric inventory (NPI-NH) [19, 20]. The NPI-NH consists of 12 subscales (e.g. apathy, anxiety) that were assessed using structured interviews. For the assessment of intervention implementation fidelity, a sum score was developed based on the frequency and duration/length of intervention components. For each delivered component one point was scored, shortcomings in duration scored 0.5 points, and zero points indicated no realization at all [18]. The baseline mean of the NPI-NH was 11, and the minimal clinically relevant intervention effect was 1. The variance between residents (0.034), was smaller than that within the residents (0.11) with an ICC of 0.24. After 19 months a reduction of some challenging behaviour categories (i.e., apathy) was measured. However, no significant changes between the control and intervention phases in the overall prevalence of at least one challenging behaviour according to NPI-NH were detected. Intervention fidelity varied between clusters in both cohorts at all time points (range: 50–100%) [21] (Fig. 1). Based on the parameters described above (incl. two cluster losses) and the final fidelity pattern of our study we estimated an average intervention effect (empirical standard error) of 0.85 (0.36) via simulation. The coverage was estimated to be 0.93, and the power was 0.66, which indicates that we could not answer the scientific question with sufficient power.

## Methods

### Simulation study

#### Study designs

**Fig. 2:**
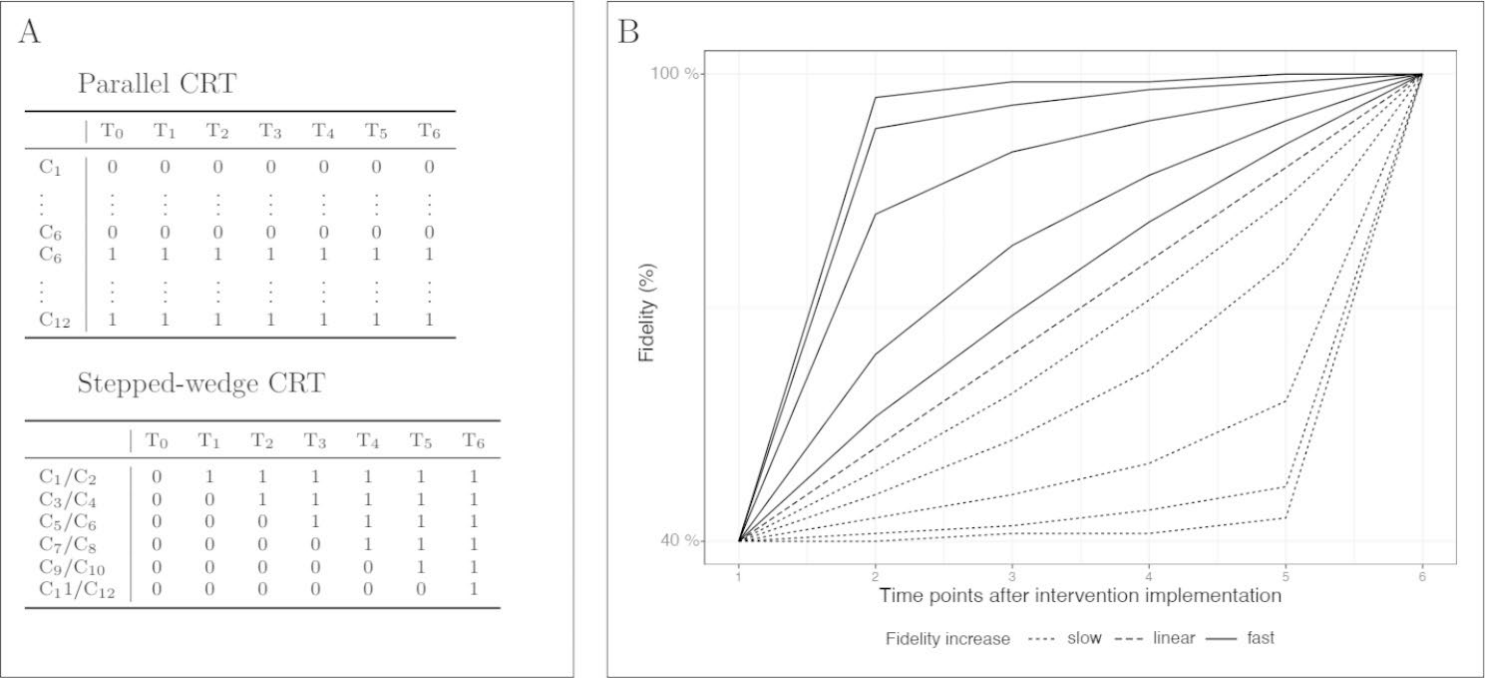
Parallel and stepped-wedge cluster randomized trial designs A: Comparison of study designs: parallel and stepped wedge designs for 12 clusters and seven time points; 1 = the intervention is implemented, 0 = the intervention is not implemented; B: Overview of potential fidelity patterns: increase of fidelity over six time points by line type (fast, linear and slow) modelled by different types of functions (logarithmic, linear, exponential)

Within implementation research, different CRT designs are commonly used. For the purpose of the simulation study, we will focus on parallel and stepped wedge CRT designs. Figure 2 A provides a schematic illustration of two comparable designs with the same number of clusters and time points using a zero to indicate the control condition and a one for the intervention condition. In parallel CRT designs, one group of clusters receives the intervention and one group the control condition over all time points. In a stepped-wedge design at each time point, some of the clusters switch from the control condition to the intervention condition.

Each of the designs has certain advantages in different situations, e.g. in case of high variation between clusters and low variation within clusters (large intraclass correlation coefficient), a stepped-wedge design requires a lower number of clusters than a parallel CRT [12]. On the other hand, a parallel CRT may require a lower total number of individuals and/or measurements than a stepped-wedge CRT [22].

For both types of study designs, several design parameters need to be specified in order to simulate data based on key features of a study: the number of clusters C, the cluster size N (the number of individuals within each cluster is assumed to be equal) and the number of time points T when outcomes of individuals are measured. In general, for effectiveness studies of interventions, these numbers are determined by sample size or power calculations prior to the study.

#### Fidelity pattern

Fidelity refers to the degree to which an intervention was implemented as it was prescribed or intended [4]. In standard clinical trials, the basic assumption is that 100% fidelity is achieved after baseline immediately after introducing the intervention and is kept constant throughout the study. This assumption does not hold in most effectiveness- implementation studies for various reasons, though. For our simulation study, we are assuming that implementation processes in real-world contexts are unfolding over time and hence, fidelity increases steadily. It needs to be acknowledged though, that in real-world studies persistent implementation challenges might occur (e.g., change of leadership team, competing projects, turnover of staff/participants) and fidelity might even decrease over time, yet we will only focus on increasing fidelity patterns in this paper. As part of this simulation, we aim to include different patterns of how fidelity might increase over time to estimate the respective effects on the power of the study. In Fig. 2B we depict hypothetical patterns of fidelity over time within a study over seven time points assuming that 100% fidelity will always be reached within the study period. Using different mathematical functions (i.e., exponential, linear, and logistical curves), we describe three prototypical patterns (slow, linear, and fast) of increasing fidelity. By considering different values for the slope parameter (for technical details see Additional file 1) we can cover a range of fidelity patterns. For our calculation within the simulation, we use fractional values ranging from 0.4 to 1.0 to depict the degree of deviation from 100% fidelity at each timepoint (i.e., for example 80% fidelity equals 0.8).

#### Simulation study

The study parameters of the FallDem example provided the basis for our simulation experiment [16, 23], values for further parameters were determined using the Shiny app for power calculations (https://clusterrcts.shinyapps.io/rshinyapp) provided by Hemming and colleagues [24]. For illustration purposes and to reduce the computational time we simplified the original study design. We aimed to optimize design parameters to achieve a power of at least 80% to detect effects under ideal circumstances (100% fidelity) for both, the stepped wedge and parallel CRT. Table 1 provides an overview of all simulation parameters. Data were simulated using the R package {samplingDataCRT} by [25]. Additional file 1 provides an overview of the simulation workflow and detailed information about the functions used. A step-by-step tutorial for the simulation including example code is provided in Additional file 2 and can be accessed online (https://github.com/INS-Basel/fidelitysim).

The simulation study explores the two study designs (parallel, stepped-wedge CRT) and hypothetical fidelity patterns (slow, linear, fast) using fixed design parameters, i.e. the number of clusters (C = 6), time points (T = 7) and patients per cluster (n = 10). For a specified set of parameters, determined by the study design and one fidelity pattern, the following two steps were replicated ten thousand times: [1] Simulation of data from the model specified by the corresponding parameters; [2] Estimation of the intervention effect using a linear mixed regression model with ’intervention’ and ’time points’ included as fixed effects in the model, and ’cluster’ included as a random effect [10]. For simplicity, a cross-sectional study type (individuals are not followed over time) was assumed. Furthermore, we limited our simulations to a continuous outcome measuring intervention effects according to our sample study.

The performance of the linear mixed model to estimate the intervention effect was then evaluated in terms of power. The empirical power was calculated as the proportion of simulation samples in which the null hypothesis of no effect (H0) is rejected at a significance level of α (usually 0.05) when H0 is false is the empirical Type II error rate. Overall, we calculated the power for 56 different combinations of different start and end values for fidelity occurring in each, parallel and stepped wedge CRT. Furthermore, we conducted a sensitivity analysis (for all 56 different combinations) to compare outcomes based on different assumptions for the ICC: 0.001/0.01 and different cluster sizes n = 10/20.

**Table 1:** Parameter settings for the simulation study

**Fidelity pattern**	
Start value	(40%, 60%, 80%)
End value	(60%, 80%, 100%)
**Design parameters**	
Design	parallel CRT, stepped-wedge CRT
Number of time points (T)	7
Number of clusters (C)	6
Number of patients within clusters (N)	10
**Model parameters**	
Overall baseline mean	Mean of outcome, measured at baseline (T0)
Intervention effect (Θ)	1
Time trend	no
Intracluster correlation coefficient (ICC)	0.001

## Results

**Fig. 3:**
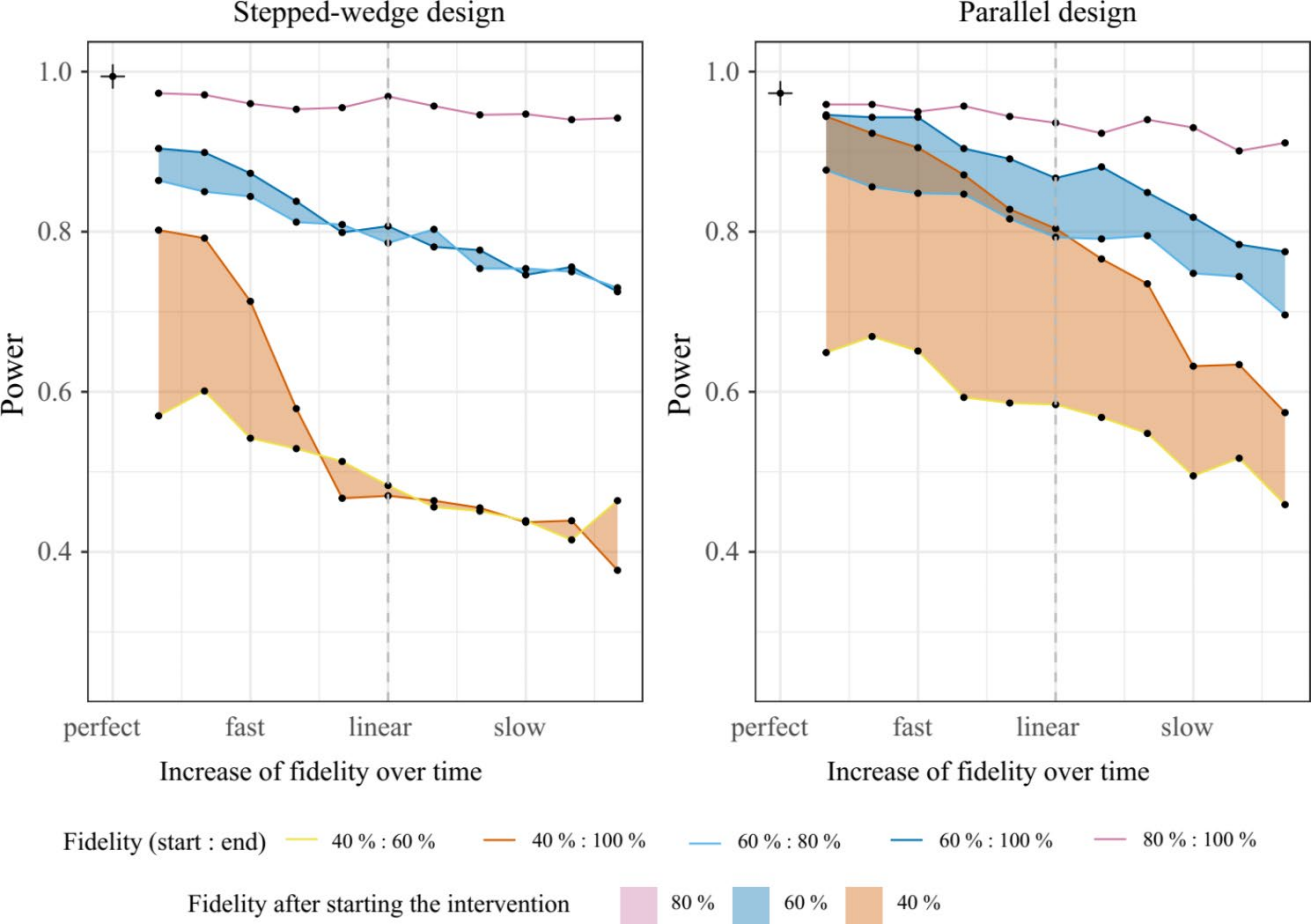
Estimated power for parallel and stepped-wedge CRT for several fidelity patterns The fidelity patterns are described by their start and end value (fidelity in % indicated by colour) and the different slopes of fidelity increase (slow, linear, fast) with in-between values. Each individual dot represents one particular fidelity pattern (i.e. specific start/end value and slope). All dots connected with a coloured line have the same start and end value but the slope of the fidelity increase is varying from fast (left) to slow (right) (Fig. 2B). The coloured areas comprise all fidelity patterns with the same start value, i.e. 40,60 or 80%. The single cross in the upper left corner represents 100% fidelity from the start

Within the parallel design the power remains comparable in simulations with the same end value for fidelity (e.g., end fidelity of 100%) and fast increase (until linear) independent from the initial value of fidelity, whereas in the stepped- wedge CRT the power is more comparable among those with the same start value of fidelity and the same increase over time independent from the initial value of fidelity. In the stepped-wedge CRT, a fidelity below 40% at the first time point after intervention rollout resulted in insufficient power (< 80%), independent of the slope of fidelity increase over time. In the parallel CRT on the other hand, the increase of the slope of fidelity over time (slow, linear, fast) shows a greater impact on effect estimation. The results of the simulation are provided in Fig. 3.

**Fig. 4:**
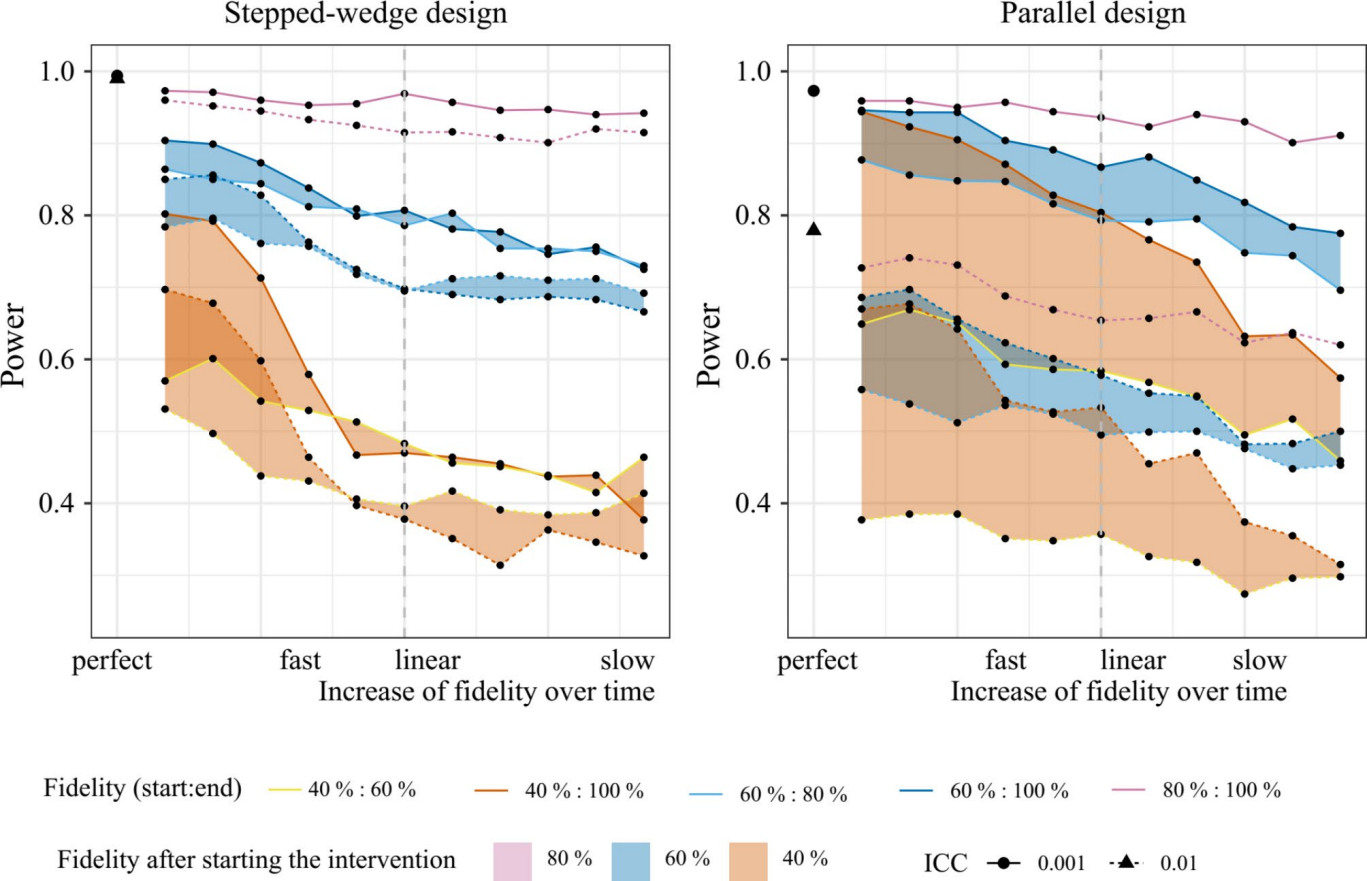
Sensitivity analysis- effect of different intra-cluster correlation coefficients (ICC) on power

**Fig. 5:**
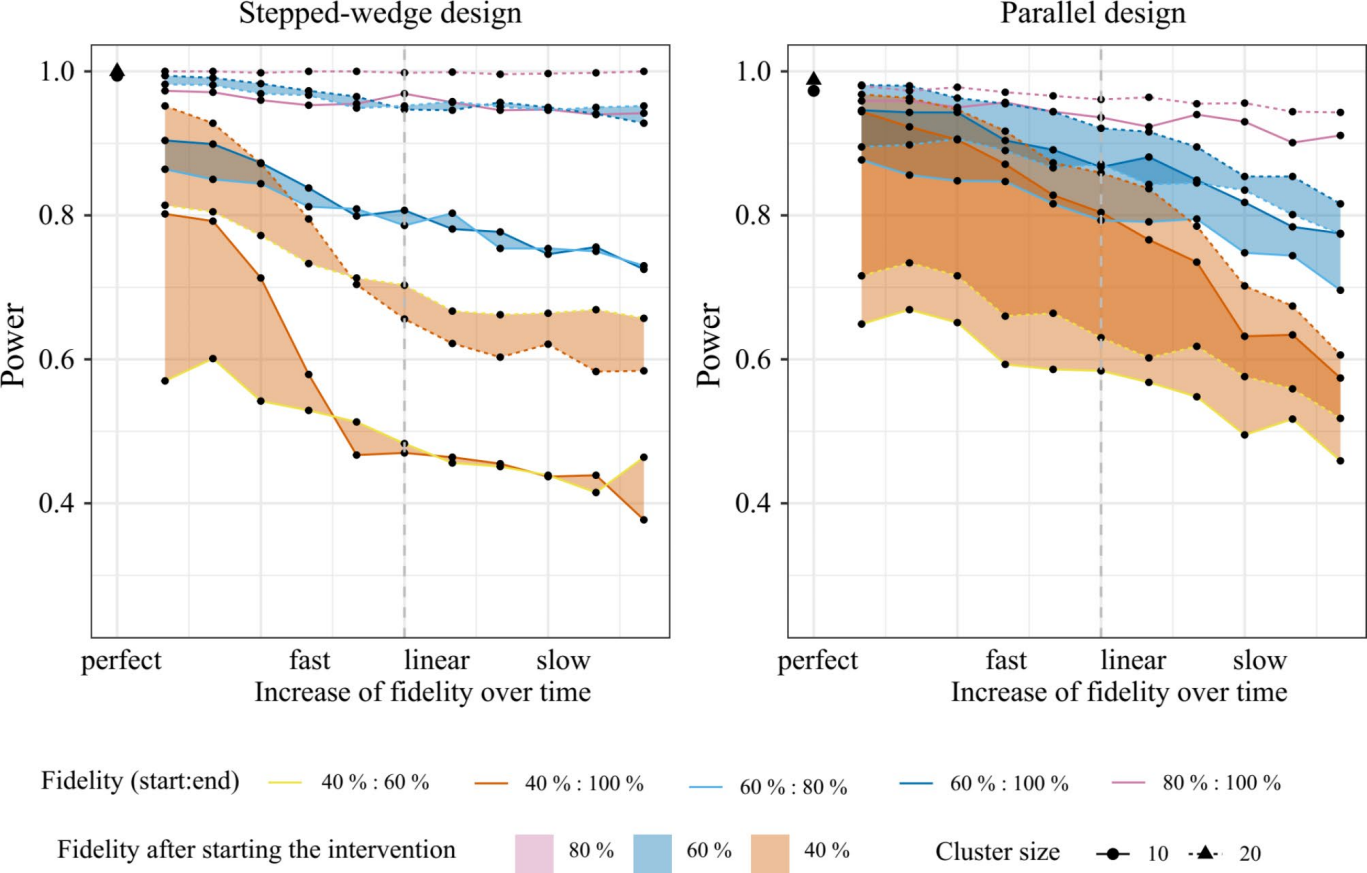
Sensitivity analysis- effect of different cluster sizes (n = 10/20) on power

Our sensitivity analysis showed that a higher ICC (0.01) leads to a general decrease of power, in particular in parallel CRT designs (Fig. 4). Here, for all potential combinations of start and end values of intervention fidelity, including 100% fidelity from the beginning, the power always remains below 80%. In stepped-wedge CRTs this effect is less pronounced. However, a higher number of individuals per cluster showed an increase in power for both study designs. Based on our simulation parameters, an increase from 10 to 20 individuals in stepped-wedge CRTs, partly increases the power of the study with overall low fidelity (i.e., start fidelity of 40% to end fidelity of 60%) from 60 to 80% depending on the slope (Fig. 5).

## Discussion

The aim of this article was to provide guidance for applied researchers conducting effectiveness-implementation hybrid trials regarding the impact of fidelity on intervention effects and power. In accordance with other studies, our findings suggest that ensuring high fidelity from the beginning is central to achieve accurate intervention effect estimates and a sufficient power of the study in stepped-wedge and parallel CRTs [26, 27]. However, we could show that in stepped-wedge CRTs the importance of high fidelity in the earlier stages is more emphasized than in parallel CRTs. In contrast, if the increase of fidelity is too slow despite relatively high starting levels, the study will likely be underpowered and the intervention effect estimates will also be biased. This effect is more accentuated in parallel CRTs, here reaching 100% fidelity within the next measurement points is imperative.

Our findings have several implications for designing and evaluating an effectiveness-implementation hybrid- trial. Before a study`s start, several general considerations about the most appropriate study design should be made based on the existing knowledge (e.g. ICC, number of clusters, cluster size, logistical aspects regarding implementation) [3, 28]. As it is often not possible to determine the patterns of fidelity prior to study start, collecting data to monitor intervention fidelity on several time points is crucial, e.g. by observations, interviews or routine data. Despite the many advantages of conducting a pilot study to get an estimation of other design parameters, e.g. ICC, information on fidelity cannot be collected prior to the real implementation.

Furthermore, our findings emphasize the importance of developing contextually-adapted implementation strategies in a preparatory phase to ensure high intervention fidelity from the beginning. Albeit in some cases fidelity might not be perfect right at start of a trial, our findings suggest that there are some options for adjustment in the trial design. One way to increase power in stepped-wedge CRTs despite low fidelity at start, is increasing the originally planned cluster size. Our findings accord with other studies that have shown that stepped-wedge CRTs gain less by adding clusters, but more by increasing the number of individuals per cluster [29]. A further approach to increase power in stepped-wedge CRTs after the study`s start, can be a prolongation of the study period since the clusters that started later might have the chance to further increase fidelity. This adjustment does not work in parallel CRTs because of the coefficient matrix for the intervention effect estimation, which is a linear combination of the observations` means in each cell. In stepped-wedge CRTs, the coefficient matrix has symmetry arising from the highest entities in the diagonal [30] and observations receive different weights. For example, in cases where fidelity is only slowly increasing, several fractional values remain close to zero resulting in a low power of the study, thus, a prolongation could increase the number of observations with high fractional values. In parallel CRTs, on the other hand, all observations receive the same weight, thus a prolongation is less effective. Despite the gains in power, it needs to be considered that additional measurement points imply higher costs and may also lead to measurement burden with only a small information gain for the overall study [31]. Overall, there are fewer options to adjust the trial design after the study has started in parallel CRT as compared to stepped-wedge CRTs, highlighting the need to determine an appropriate and powerful design before the start.

Effectiveness-implementation hybrid studies aim to accelerate the translational process of efficacious interventions to practice settings. Determining effectiveness in real-world settings with low internal validity is a well-known challenge therefore it is crucial to take contextual information and implementation outcomes into account. Fidelity is a central implementation outcome with regard to effectiveness however its assessment can be a challenge in complex interventions due to its numerous components that are partly interrelated. Developing a logic model can help to disentangle effects prospectively and allows planning rigorous evaluation [32]. For the assessment of fidelity, it is necessary to distinguish between core functions of intervention, i.e., core purpose/mechanism of change and form, i.e. specific strategies or activities that are necessary to carry out the intervention. While the form of the intervention can be adapted to the local context and the needs of the respective population - fidelity to the core functions of the intervention is essential [6]. From an implementation science perspective, the ideal approach to increase fidelity is the modification of implementation strategies or the intervention. To ensure reproducibility and comparability, modifications should be documented throughout the effectiveness- implementation trials using appropriate tools [33, 34]. A process evaluation alongside the implementation trial using quantitative and qualitative data is central to gain an understanding of the implementation effectiveness and to conclude data analysis [32, 35]. Once fidelity is known for all time points, a posthoc analysis can shed light on the final intervention effect. For this, fractional values between 0 and 1, reflecting the degree of fidelity at different time points can be added to the statistical model [26].

In the literature, only a few effectiveness studies consider the effect of fidelity on the intervention effect, in particular in hybrid studies. Yet, our findings highlight that the effect of fidelity on outcomes is not to be neglected and further research into study designs accounting for potential deviations is needed. One promising approach that should be further explored in the implementation science paradigm is sequential multiple assignment randomized trial (SMART) designs [36]. SMART designs originated from research on adaptive treatment strategies where the treatment intensity and type are adjusted according to the individual response. The same principle can be applied to implementation strategies aiming to increase fidelity as showcased in the example of Kilbourne and colleagues [37].

This study`s strengths include the combination of data from a practical example and simulations to derive recommendations for applied researchers to deal with fidelity in the two most common study designs, stepped-wedge and parallel CRTs. A further strength of this study is the available simulation code provided as a tutorial, allowing other researchers to investigate the impact of various fidelity patterns on their study`s power. In addition to its strengths, the limitations of this study need to be recognized. First, in general, a simulation provides a simplification of the ’real world’ and can provide only an estimation as good as the specified parameters. The results of the simulation are based on linear mixed-effects models, which are primarily used for continuous outcomes. Hence, results might differ when using generalized linear mixed models for binary outcomes. Furthermore, within our simulation, not all common design features were covered. This was partly due to computational (time) limits, but primarily to highlight the focus on the application in conjunction with the clinical study example. In this manuscript we focused on patterns of increasing fidelity, as implementation strategies aim to improve the implementation of evidence, and consequently, increase fidelity. Unfortunately, decreasing fidelity can often be observed in many implementation projects. Our simulation code and tutorial allow researchers to explore decreasing patterns or combinations of increasing and decreasing patterns.

## Conclusions

Effectiveness-implementation hybrid designs present a valuable approach to concurrently determine the effectiveness of intervention and implementation strategies. Parallel and stepped-wedge CRTs both are common study designs in effectiveness-implementation trials, yet they have different properties to adapt to real-world influences. This study discusses the importance of intervention fidelity for the study`s power and highlights different recommendations to deal with low fidelity in parallel and stepped-wedge CRTs from a design perspective.

## Electronic supplementary material

Below is the link to the electronic supplementary material.

Supplementary Material 1

Supplementary Material 2

## Data Availability

The dataset used and/or analysed during the current study is available from the corresponding author on reasonable request.

## List of Abbreviations

CRT: Cluster randomized trial
ICC: Intracluster correlation coefficient
NH: Nursing home
SMART: Sequential multiple assignment randomized trial
