# Supplementary material for: The unrecognized role of fidelity in effectiveness-implementation hybrid trials: simulation study and guidance for implementation researchers
Source: BMC Med Res Methodol. 2023 May 13;23:116. doi: 10.1186/s12874-023-01943-3 (PMC10183132; doi:10.1186/s12874-023-01943-3)
Supplement: Supplementary file 1 — Supplementary Material 1 [file 12874_2023_1943_MOESM1_ESM.pdf]

The unrecognized role of fidelity in hybrid effectiveness-implementation trials: simulation study and guidance for implementation researchers

Diana Trutschel [ORCID: 0000-0002-1727-9736]
Catherine Blatter [ORCID: 0000-0002-4069-7178]
Michael Simon [ORCID: 0000-0003-2349-7219]
Thekla Brunkert [ORCID: 0000-0002-0369-1826]

2022-10-30T00:00:00+02:00

Table of contents

Preface 3

Citation . . . 3

1 Introduction 4

1.1 Required R packages . . . 4

1.2 Other considerations . . . 5

2 Step-by-step simulation 6

2.1 Simulation experiment for one setting . . . 7

2.1.1 Study design . . . 8

2.1.2 Assumptions about fidelity pattern . . . 9

2.1.3 Specifying model parameter . . . 11

2.1.4 Defining number of iterations for the simulation . . . 12

2.1.5 Simulation experiment . . . 12

2.2 Comparison of several Fidelity patterns . . . 13

3 Detailed statistical information 17

3.1 Explanation of simulation experiment and provided corresponding function . . 17

3.1.1 Determining the design matrix regarding the chosen design . . . 17

3.1.2 Sampling data of a cluster randomized trial with a given design . . . 18

3.1.3 Effect estimation from the data . . . 21

3.2 Determine differrent design matrices . . . 21

3.2.1 Parallel cluster randomized trials . . . 22

3.2.2 Stepped wedge cluster randomized trial . . . 22

3.3 Determine different fidelity patterns . . . 23

3.3.1 Slow increase of fidelity using exponential function . . . 25

3.3.2 Linear increase of fidelity using linear function . . . 26

3.3.3 Fast increase of fidelity using logarithmic function . . . 27

References 29

Preface

This online tutorial serves as a hands-on guide for researchers to replicate the simulation described in

[The unrecognized role of fidelity in hybrid effectiveness-implementation trials: simulation study and guidance for implementation researchers].

This supplementary material was designed to support applied researchers to adapt the simulation with required parameters for their own study. Consequently, the content is split in the following chapters:

- Chapter 1 gives an overview of the technical requirements
- Chapter 2 describes the step-by-step approach how to conduct the simulation for a specific research study, based on parallel or stepped wedge CRTs, and furthermore shows the comparison of several fidelity patterns
- Chapter 3 provides optional statistical information (for a detailed understanding of the method)

Citation

If you find this work helpful, please cite both the online tutorial and the paper by the reference of the original paper as in:

[our paper]

1 Introduction

The following technical setup is needed to pursue a local simulation on your computer:

- A local installation of the R statistical programming language (R Core Team 2022) is required.
  The current version can be downloaded from https://cran.r-studio.org.
- In addition, we recommend the use of RStudio Desktop (an integrated development environment) which provides an excellent user interface for working with R.
  It is available from here: https://www.rstudio.com/products/rstudio/download/.

Both R and RStudio (as well as further used R packages) are free software.

To follow this tutorial, we assume basic familiarity with the use of the R language.

1.1 Required R packages

The following R packages are needed to run the simulation:

- {fidelitysim} (Trutschel and Blatter 2022) - holds packaged code for the simulation experiment

It uses and builds upon:

- {samplingDataCRT} v1.0 (Trutschel and Treutler 2017) - for sampling data matrices
- {lme4} (Bates et al. 2015) - for linear model estimation
- {ggplot2} (Wickham 2016) - for visualizing the results

You can install the necessary packages by:

```r
# to install {fidelitysim} we need the {remotes}-package
install.packages("remotes")

# then install {fidelitysim} from GitHub
remotes::install_github("INS-Basel/fidelitysim")
```

The other packages are installed from CRAN:

```
# install packages from CRAN
install.packages(c("samplingDataCRT", "lme4", "ggplot2"))
```

Consequently, to start the calculation the package needs to be loaded by:

```
library(fidelitysim)
```

1.2 Other considerations

The local simulation can - depending on the number of clusters and the computational power - take up to 10-12 hours when the commenly recommended number of iterations of 10,000 is used. We suggest the user to start own simulations with a small number and than increase, when it is clear the code is running.

2 Step-by-step simulation

The following chapter is separated in two parts:

1. Simulation experiment for one setting by choosing between
    - Parallel or stepped wedge designs
    - Studies with different fidelity patterns
2. Simulation experiments to compare of several fidelity patterns

Figure 2.1 provides an overview of the simulation workflow.

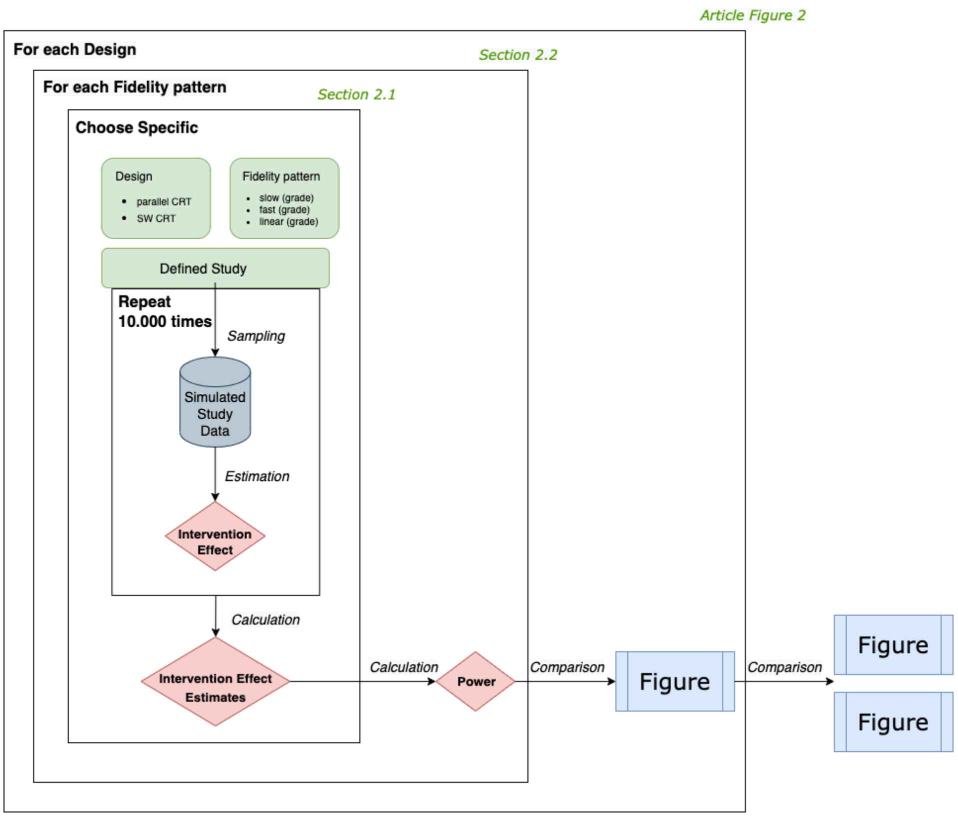

Figure 2.1: Overview of simulation workflow

2.1 Simulation experiment for one setting

According to the inner frame of Figure 2.1, several specifications have to be made to evaluate the performance of a chosen design and fidelity pattern.

Within each simulation-experiment, the steps *data sampling* and *effect estimation* will be repeated (further explanation see Chapter 3.) To simplify the process of the whole simulation-experiment, we packaged the code into the R package {fidelitysim}.

The function `fidelitysim::simulation()` includes the steps: data sampling for a specific design and existing implementation error, effect estimation, performance measures calculation and the following parameters are needed as arguments to the function:

- number of repeats for the simulation
- chosen design (including study design, number of cluster, time points and individuals per cluster)
- model parameters (effect, variances, …)
- two design matrices which indicates whether deviation from perfect situations are assumed or not

The outputs of the function are:

- effect estimates
- performance measures

After repeating the steps of the simulation the obtained effect estimates can be evaluated for the performance of the model. The following performance measures of a simulation are provided:

- Bias: mean deviation of the estimates from the true value of the parameter of interest (intervention effect) is an indicator of accuracy

- Coverage: measurement used to control the Type I error rate for testing the null hypothesis (H0) of no effect

- Power: proportion of simulation samples in which the H0 of no effect is rejected at a significance level of α when H0 is false (is related to the empirical Type II error rate).

In the following, we will show an example of a whole simulation experiment for a parallel cluster randomized trial. Further we will point out necessary adaptions for stepped-wedge designs (regarding the parameter setting).

First, make sure that necessary packages are loaded (and to obtain the same results) indicate a seed for the simulation:

```
# load necessary packages
library(fidelitysim)
library(samplingDataCRT)

# seed
set.seed(1234)
```

One setting includes the specification of:

1. Study design
2. Assumptions about fidelity pattern
3. Model parameter
4. Iterations of the simulation

and finally,

5. the simulation experiment can be performed.

2.1.1 Study design

Based on the study setup, the following parameters for the study design need to be defined:

- No. of clusters (e.g hospitals)
- No. of individuals per cluster (and time point) as the cluster size
- No. of time points the cluster were followed
- Design type controls if individuals will be followed over time (cross-sectional or longitudinal): cross-sectional type, which indicates trials where individuals within the cluster can change over time points (however this design indicator is not necessary for conducting the design matrix)

A design matrix according the study design has to be determined by the function `designMatrix()`. Here, two arguments are specific for the different types of studies. If the `design = 'parallel'`, then the parameter `nSW` indicates the number of clusters as being the control group. If choosing `design = 'SWD'` (which is also set by default), the parameter `nSW` indicates the number of cluster-switches per time point from control to intervention group (which highly dependent on the number of clusters and time points).

For our example we will use the following parameter:

- Number of clusters: 6
- Cluster size: 10

- Number of time points: 7
- Cross-sectional design

and 3 clusters are in the control and 3 clusters in the intervention group.

In the next step we determine the corresponding design matrix for a parallel cluster randomized study with this specific set of design parameters as follows:

```
## Design matrix ##

I <- 6       # number of clusters
J <- 10      # number of individuals per cluster (used later in simulation-step)
K <- 7       # number of time points
Sw <- 1      # number of cluster switches per time point can be manually set
type <- "cross-sec" #Cross-sectional design, indicates that
#individuals within the cluster can change over time points

# design matrix for parallel design
(designMat_prll <- designMatrix(
  nC = I, nT = K, nSw = round(I / 2),
  design = "parallel"
))
```

```
     [,1] [,2] [,3] [,4] [,5] [,6] [,7]
[1,]    0    0    0    0    0    0    0
[2,]    0    0    0    0    0    0    0
[3,]    0    0    0    0    0    0    0
[4,]    1    1    1    1    1    1    1
[5,]    1    1    1    1    1    1    1
[6,]    1    1    1    1    1    1    1
```

2.1.2 Assumptions about fidelity pattern

The following step is a central part of this tutorial. We want to examine different fidelity patterns and their implications for the study effects.

To simulate a specific fidelity pattern, a second design matrix indicating the pattern of fidelity has to be provided and indicates if there are deviations from the perfect situation (100% fidelity) present or not. For the simulation we have to add fractional values according to the chosen fidelity pattern. There are 3 functions within the R-package {fidelitysim} that can be used to specify several fidelity patterns. The patterns can be specified based on a slow, linear or fast increase and the respective start and end value for fidelity. For further explanation of fidelity patterns see Section 3.3. The selected fidelity patterns can then be pushed to the design matrix with the R-package {samplingDataCRT}, for more explanation see Section 3.2.

For our example we use the same design parameters, i.e. parallel cluster randomized trial, 6 timepoints, etc. and assume a linear increase of fidelity from 40% to 80% from the first measurement point after introduction of the intervention to the final measurement point (time point `K`). Based on these parameters we will create a new design matrix `X.A` that incorporates fractional values based on the provided fidelity pattern. This new design matrix will be used as a reference matrix in our simulation.

3 arguments have to be specified to determine the fidelity pattern using the provided function `find.Fidelity.linear`:

```
### Fidelity parameters ###

# Fidelity at the begin
Fid.T1 <- 0.4
# Fidelity at the end
Fid.End <- 0.8

# slope for linear function
m <- (Fid.T1 - Fid.End) / (1 - (K - 1))
```

```
# model linear increase
res.lin <- find.Fidelity.linear(time.points = K, Fid.End, Fid.T1)
```

(The application of the other two functions for slow (exponential) and fast (logarithmic) increase are shown in Section 3.3.)

Then, we create the new design matrix using the function `implemMatrix.parallel` :

```
# design matrix of a linear fidelity pattern
(fidelMat_prll <- implemMatrix.parallel(
  nC = I, nT = K, nSw = round(I / 2),
  pattern = res.lin[, "Fidelity.Prozent"] / 100
))
```

```
     [,1] [,2] [,3] [,4] [,5] [,6] [,7]
[1,]  0.0 0.00 0.00  0.0 0.00 0.00  0.0
[2,]  0.0 0.00 0.00  0.0 0.00 0.00  0.0
[3,]  0.0 0.00 0.00  0.0 0.00 0.00  0.0
[4,]  0.4 0.47 0.53  0.6 0.67 0.73  0.8
[5,]  0.4 0.47 0.53  0.6 0.67 0.73  0.8
[6,]  0.4 0.47 0.53  0.6 0.67 0.73  0.8
```

This matrix reflect the fidelity pattern including the implementation error (deviation form 100%). Both design matrices are later used for the simulation as arguments `X = designMat_prll` and `X.A = fidelMat_prll` for simulation Section 2.1.5. In a *perfect* situation - where we assume 100% fidelity at every time point, both matrices are the same.

2.1.3 Specifying model parameter

In addition to the design parameters, we have to define the following model parameters as well:

- Baseline mean of the outcome of interest (e.g. mean quality of life score) μ_0
- Intervention effect (the change of scores after intervention) Θ
- Intra-cluster correlation coefficient ICC (between cluster and error variance σ_c, σ_e)

- If applicable: time trend (effect of each time point during followed time points)

For our example we use the following model parameter:

- baseline mean = 10
- intervention effect = 1
- no time trend
- ICC of 0.001

```
## Model parameter ##

mu.0 <- 10 # Baseline mean of the outcome of interest
theta <- 1 # Intervention effect
betas <- rep(0, K - 1) # no time trend, but could be included

# variability within or error variance (H&H sigma)
sigma.1 <- 2
# variability within clusters, if longitudina data
sigma.2 <- NULL
# between clusters variability (H&H tau)
sigma.3 <- sigma.1 * sqrt(0.001 / (1 - 0.001))

# resulting ICC
(ICC <- sigma.3^2 / (sigma.3^2 + sigma.1^2))
```

```
[1] 0.001
```

When choosing a longitudinal instead of a cross-sectional design a third variance needs to be specified (see for more detail into Section 3.1.2).

2.1.4 Defining number of iterations for the simulation

In addition to the parameters set above, we need to define the number of iterations for the simulation. The number determines how often the sampling and estimation should be repeated to finally calculate the performance measures. In the literature 10,000 and 100,000 iterations are recommended to obtain valid results, however, this validity comes at a time cost. In our example we will set n = 1,000 iterations with regard to the computing time (and can be set by the user under consideration of their machine's computational power).

```
## Number of iterations of the simulation ##
anzSim <- 1000
```

2.1.5 Simulation experiment

Since we set all necessary arguments for the usage of the provided simulation function:

1. Study design
2. Model parameter
3. Indication if there are deviations from perfect situations or not
4. Iterations of the simulation

we can start the simulation by using the provided function `simulation`:

```
# linear increase of fidelity
res.Simu.parallel.lin <- simulation(
  anzSim = anzSim, #Simulation parameter
  type = type, K = K, J = J, I = I, #design parameter
  sigma.1 = sigma.1, sigma.3 = sigma.3, #model parameters
  mu.0 = mu.0, theta = theta, betas = betas, #model parameters
  X = designMat_prll, X.A = fidelMat_prll #design matrices
)
```

For this design setting and number of iteration (n=1000), a computational time of 32.53 min is needed

The output of the simulation provides a total of n =22 results, among the following performance parameters (see also Section 2.1):

- Bias
- Coverage
- Power

The mean estimate of the intervention effect from all iterations and the power of the design can be accessed like this:

```
# mean estimated intervention effect
round(res.Simu.parallel.lin["intervention Mean ."],3)
```

```
intervention Mean .
              0.615
```

```
# estimated power
round(res.Simu.parallel.lin["Power.Intervention"],3)
```

```
Power.Intervention
             0.738
```

We obtain a power of 0.738 to detect an intervention effect of 1 within an parallel study with 6 clusters followed 7 time points and a cluster size of 10 when an linear increase of the fidelity from 40% to 80% is achieved until end of the study.

2.2 Comparison of several Fidelity patterns

To compare the effect of different fidelity patterns, a repetition of the simulation has to be conducted.

We will continue with our example from before (see Section 2.1.1 - Section 2.1.3) and examine in total seven different fidelity patterns. We will assume (A) three types of slow increases, (B) one linear increase and (C) three types of fast increases of fidelity for this design setting (see Section 3.3 for further explanation) and compare with the perfect situation.

```
### several Slopes indicating the degree of increase ###
slope.seq<-round(exp(1)^(seq(-2,2,2)),2)
nr.sl<-length(slope.seq)
```

At first, perfect situation simulation (no deviation from 100% implementation).

```
################################
# perfect implementation
# no individual or cluster miss
################################
```

```
#design matrix of perfect situation
X<-samplingDataCRT::designMatrix(nC=I, nT=K, nSw=round(I/2), design="parallel")
res<-fidelitysim::simulation(
                  anzSim=anzSim, #Simulation parameter
                  type="cross-sec", K=K,J=J,I=I, #design paramter
                  sigma.1=sigma.1,sigma.3=sigma.3, #model parameters
                  mu.0=mu.0, theta=theta,betas=betas,
                  X=X, X.A=X #design matrices
                  )
res<-as.data.frame(t(res))
```

Next for (A) several slow increase (reflected by an exponential function):

```
###all the other patterns
res.Simu<-data.frame()

#exponential increase
for(sl in 1:nr.sl){#for each slope

   #Fidelity pattern
    res.exp<-fidelitysim::find.Fidelity.exp(time.points=K,
                               Fid.End, Fid.T1,
                               par.slope=slope.seq[sl])
    #new design matrix
    A1.exp <-fidelitysim::implemMatrix.parallel(nC=I, nT=K, nSw=round(I/2),
                               pattern=res.exp[,"Fidelity.Prozent"]/100)
     #simulation experiment
    res<-fidelitysim::simulation(anzSim=anzSim,
                    type="cross-sec",  K=K,J=J,I=I,
                    sigma.1=sigma.1,sigma.3=sigma.3,
                    mu.0=mu.0, theta=theta,betas=betas,
                    X=X, X.A=A1.exp
                    )
    res<-as.data.frame(t(res))
    #save results
    res.Simu<-rbind(res.Simu,
                   data.frame( res,
                      D="exp", slope=slope.seq[sl], sort=2+nr.sl+(nr.sl-sl+1),
                      Fid.Begin=Fid.T1, Fid.END=Fid.End)
    )

}
```

For (B) linear increase:

```
##linear increase
m<-(Fid.T1-Fid.End)/(1-(K-1))
#Fidelity pattern
res.lin<-fidelitysim::find.Fidelity.linear(time.points=K, Fid.End, Fid.T1)
#ne design matrix
A1.lin <-fidelitysim::implemMatrix.parallel(nC=I, nT=K, nSw=round(I/2),
                              pattern=res.lin[,"Fidelity.Prozent"]/100)
#simulation experiment
res<-fidelitysim::simulation(anzSim=anzSim,
                type="cross-sec", K=K,J=J,I=I,
                sigma.1=sigma.1,sigma.3=sigma.3,
                mu.0=mu.0, theta=theta,betas=betas,
                X=X, X.A=A1.lin)
res<-as.data.frame(t(res))
```

For (C) several fast increase (reflected by a logarithmic function):

```
###all the other patterns
res.Simu<-data.frame()

#logistic increase
for(sl in 1:nr.sl){#for each slope
    #Fidelity pattern
    res.log<-fidelitysim::find.Fidelity.log(time.points=K,
                              Fid.End, Fid.T1,
                              par.slope=slope.seq[sl])
    #new design matrix
    A1.log <-fidelitysim::implemMatrix.parallel(nC=I, nT=K, nSw=round(I/2),
                              pattern=res.log[,"Fidelity.Prozent"]/100)
    #simulation experiment
    res<-fidelitysim::simulation(anzSim=anzSim,
                type="cross-sec", K=K,J=J,I=I,
                sigma.1=sigma.1,sigma.3=sigma.3,
                mu.0=mu.0, theta=theta,betas=betas,
                X=X, X.A=A1.log)
    res<-as.data.frame(t(res))
    #save results
    res.Simu<-rbind(res.Simu,
               data.frame( res,
                  D="log", slope=slope.seq[sl], sort=1+sl,
```

```
                                    Fid.Begin=Fid.T1, Fid.END=Fid.End)
    )
  }
```

Figure 2.2 summarizes the results of the simulation for different fidelity patterns regarding power of the study. The results shown are performed by 10,000 iterations for the simulation to have precise estimates. For this design setting and number of iteration (n=10,000), a computational time of 7 hours is needed.

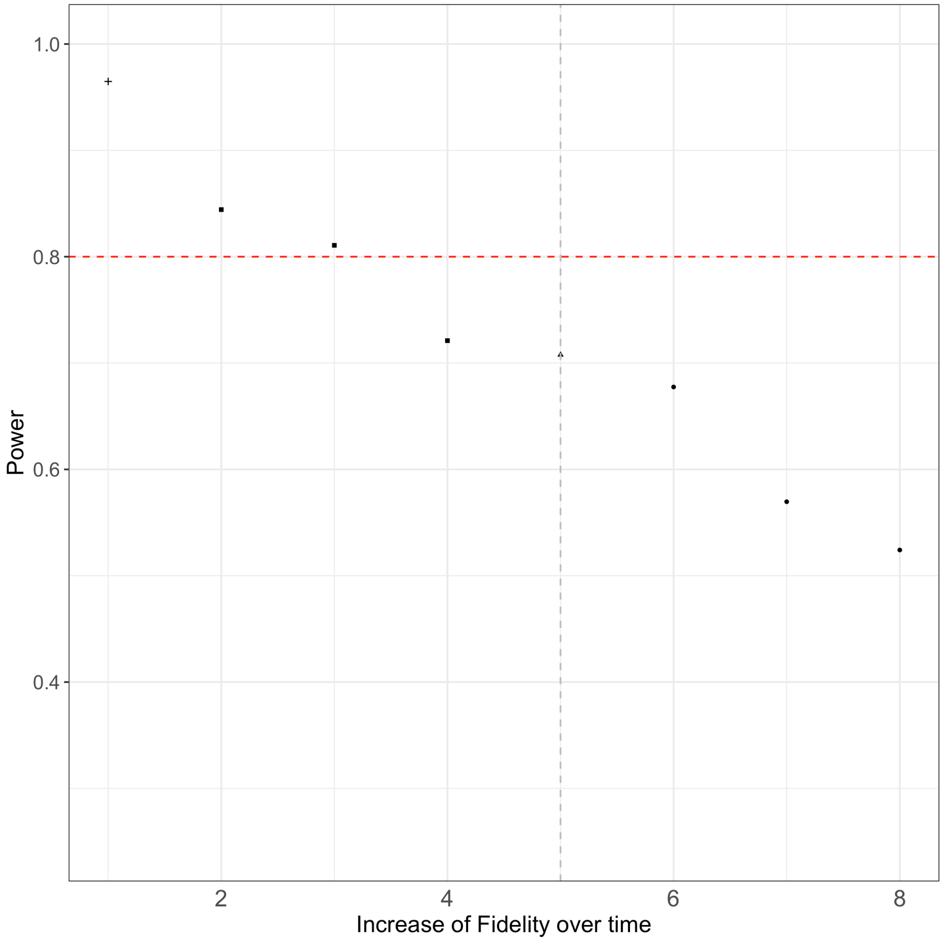

Figure 2.2: Results of simulation for several fidelity pattern scenarios

3 Detailed statistical information

This chapter offers additional statistical information.

1. Steps within one simulation experiment
2. How to conduct design matrices
3. How to determine fidelity patterns and corresponding design matrices

3.1 Explanation of simulation experiment and provided corresponding function

For a simulation experiment it is not necessary to understand the whole statistical modeling background, which is figured out here.

Each simulation step includes three necessary steps:

- Determining the design matrix regarding the specified design
- Sampling data
- Effect estimation from the data

and will be explained with more detail in the following subsections.

For the first two steps, the R-package {samplingDataCRT} v1.0 (Trutschel and Treutler 2017) is needed, for the last {lme4} (Bates et al. 2015). The first step is done once and the latter two are used repeatedly through one simulation experiment within each scenario.

3.1.1 Determining the design matrix regarding the chosen design

To specify a cluster randomized study the following parameter has to be determined:

- Number of clusters (e.g. hospitals, nursing homes, …) obtained through the study
- Number of time points the clusters are followed
- Cluster size refers to the number of individuals obtained within one cluster
- Study design: parallel design or Stepped wedge design (cross-over as well possible)
- Study type: cross-sectional if individuals could be different within the cluster between time points or longitudinal if individuals are followed over time

Based on the example in the main article, we specify a hypothetical example including parameter settings for a reference setup of the simulation experiment: *a cross-sectional stepped wedge cluster randomized trial with 6 nursing homes, 7 time points and 10 individuals* within each nursing home and time point (see main article). All parameters can be adapted to an own practical example. The resulting reference design matrix can be create manually (by create a corresponding matrix) or by `designMatrix()`.

```
#####################################################
#using the parameter setting of Table 1 in the article
#####################################################

## Design ##
############
K<-7 #number of time points
I<-6 #number of cluster
Sw<-1 #number of cluster switches per time point can be manually set
J<-10 #Subjects =  Number of individuals per cluster

#design matrix of "SWD" with given setting
(X<-samplingDataCRT::designMatrix(nC=I, nT=K, nSw=Sw))
```

```
     [,1] [,2] [,3] [,4] [,5] [,6] [,7]
[1,]    0    1    1    1    1    1    1
[2,]    0    0    1    1    1    1    1
[3,]    0    0    0    1    1    1    1
[4,]    0    0    0    0    1    1    1
[5,]    0    0    0    0    0    1    1
[6,]    0    0    0    0    0    0    1
```

3.1.2 Sampling data of a cluster randomized trial with a given design

To sample data for a cluster randomized trial using sampling from a **multivariate normal distribution** provided by the package {samplingDataCRT}, several model parameter has also be specified:

- Baseline mean of the outcome of interest
- Intervention effect (change/difference in mean outcome) by implementing the intervention)
- Time trend effects
- Variance within the multilevel data: between clusters, between individuals, within individuals which provide an estimate of the intra-cluster correlation coefficient (ICC) as an measure of dependencies within clusters.

In the example here we set the mean outcome to 10 (e.g. the mean population value of measured quality of life), the intervention effect which aimed to change quality of life of 1, and no time trends. With a given between cluster variance of `sigma.3` and a error variance of `sigma.1`, the resulting ICC is `ICC`.

```
## Model parameter ##
####################
mu.0<- 10            # Baseline mean of the outcome of interest
theta <- 1           # intervention effect
betas<-rep(0, K-1)   # no Time trend, but could be included

# variability within or error variance (H&H sigma)
sigma.1<-2
# variability within clusters, if longitudinal data
sigma.2<-NULL
# between clusters variability (H&H tau)
sigma.3<-sigma.1*sqrt(0.001/(1-0.001))

#resulting ICC
(ICC<-sigma.3^2/(sigma.3^2+sigma.1^2))
```

```
[1] 0.001
```

To note, by choosing **longitudinal or cross-sectional design**, we need to specify in the first case 3, in the second only two **variances** regarding using a **three- instead of a two-level hierarchical experiment** with the following meaning:

- three-level design (longitudinal data):
  - between clusters variability
  - within cluster (or between individuals) variability
  - within individuals (or error) variability
- two-level design (cross-sectional data):
  - between clusters variability σ (Hussey and Hughes 2007)
  - within cluster (or error) variability τ (Hussey and Hughes 2007)

A complete data set for a special design with a given setup can be sampled by the function `sampleData()`. Therefore,the complete data design matrix and the covariance-variance matrix for the data given the design are also needed to be specified with `completeDataDesignMatrix()` and `CovMat.Design()`. The complete data design matrix has the size of (Number of cluster x cluster size x Number of time points) rows and (Number of model parameters) columns. To sample the data from for a cluster randomized trial, it has

additionally be specified, if the individuals are followed over time (longitudinal design) or not (cross-sectional design).

```
#complete data design matrix
D<-samplingDataCRT::completeDataDesignMatrix(J, X)
(dim(D))
```

```
[1] 420   8
```

```
#covariance-variance matrix for the data given the design
V<-samplingDataCRT::CovMat.Design(K, J, I, sigma.1=sigma.1, sigma.3=sigma.3)
dim(V)
```

```
[1] 420 420
```

```
#corresponding fixed effects in linear mixed model
parameters<-c(mu.0, betas, theta)

#sample complete data given the setup
# study design type = cross-sectional
type<-"cross-sec"
sample.data<-samplingDataCRT::sampleData(type = type, K=K,J=J,I=I,
                                          D=D, V=V, parameters=parameters)
```

To validate the number of observations provided by the sampling method a summary of the data can be conducted.

```
dim(sample.data)
```

```
[1] 420   5
```

```
#show the number of observations within the SWD
xtabs(~cluster+measurement, data=sample.data)
```

```
       measurement
cluster  1  2  3  4  5  6  7
      1 10 10 10 10 10 10 10
      2 10 10 10 10 10 10 10
```

```
3 10 10 10 10 10 10 10
4 10 10 10 10 10 10 10
5 10 10 10 10 10 10 10
6 10 10 10 10 10 10 10
```

3.1.3 Effect estimation from the data

The sampled data can then be analyzed by a **linear mixed model** with `lme4::lmer()`, where the parameter of the model will be estimated.

```
#analysis of the two-level data by a linear mixe model
lme4::lmer(val~intervention+measurement + (1|cluster), data=sample.data)
```

```
Linear mixed model fit by REML ['lmerMod']
Formula: val ~ intervention + measurement + (1 | cluster)
   Data: sample.data
REML criterion at convergence: 1480.436
Random effects:
 Groups   Name        Std.Dev.
 cluster  (Intercept) 0.246
 Residual             1.393
Number of obs: 420, groups:  cluster, 6
Fixed Effects:
 (Intercept)  intervention  measurement2  measurement3  measurement4
     9.95830       1.05820       0.03535       0.37739       0.58820
measurement5  measurement6  measurement7
     0.06163       0.50931      -0.02286
```

This process of sampling data and effect estimation from the data will be used repeatedly through the simulation experiment within each scenario setting.

3.2 Determine differrent design matrices

The function `designMatrix()` of the R-package {samplingDataCRT} provides the design matrix of cluster randomized trials with several study designs, given the number of clusters, time points and cluster size assumed for the trial. We show here for two examples:

- parallel
- SWD

3.2.1 Parallel cluster randomized trials

Within the function the `design` argument is set to 'parallel',
and the parameter `nSW` indicates the number of clusters that are being control group.

```
## Design paramter ##
######################
I <- 6 # number of clusters
J <- 10 # number of individuals per cluster (used later in simulation-step)
K <- 7 # number of time points
Sw <- round(I / 2) # number of cluster within the control group

# design matrix for parallel design
(designMat_prll <- samplingDataCRT::designMatrix(nC = I, nT = K, nSw = round(I / 2),
                                                 design = "parallel"))
```

```
     [,1] [,2] [,3] [,4] [,5] [,6] [,7]
[1,]    0    0    0    0    0    0    0
[2,]    0    0    0    0    0    0    0
[3,]    0    0    0    0    0    0    0
[4,]    1    1    1    1    1    1    1
[5,]    1    1    1    1    1    1    1
[6,]    1    1    1    1    1    1    1
```

3.2.2 Stepped wedge cluster randomized trial

Whereas the design parameters can be the same as in the parallel study the design matrix differs accordingly when changing the argument `design = "SWD"` (which is also set by default, so it has not to be specified). The parameter 'nSW' indicates here the number of cluster switches per time point from control to intervention group.

```
## Design matrix ##

I <- 6 # number of clusters
J <- 10 # number of individuals per cluster (used later in simulation-step)
K <- 7 # number of time points
Sw <- 1 # number of cluster switches per time point can be manually set

# design matrix of "SWD"
(designMat_SWD <- samplingDataCRT::designMatrix(nC = I, nT = K,
                                                nSw = Sw, design = "SWD"))
```

```
     [,1] [,2] [,3] [,4] [,5] [,6] [,7]
[1,]    0    1    1    1    1    1    1
[2,]    0    0    1    1    1    1    1
[3,]    0    0    0    1    1    1    1
[4,]    0    0    0    0    1    1    1
[5,]    0    0    0    0    0    1    1
[6,]    0    0    0    0    0    0    1
```

3.3 Determine different fidelity patterns

Fidelity refers to the degree to which an intervention was implemented as it was prescribed or intended. We aim to include different patterns of how fidelity might increase over time to estimate the respective effects on power of the study. To describe hypothetical fidelity patterns of increasing fidelity (slow, linear, fast) different mathematical functions (i.e. logistic, linear and exponential curves) are implemented. By considering different values for the slope parameter we can cover a range of fidelity patterns. The slope parameter ranges form $(0, \infty)$, where a slope parameter near to 0 indicates a increase far away from a linear (fast increase upper left corner, slow increase right bottom corner curve) and a great slope parameter near to linear (see Figure 3.1).

```r
library(ggplot2)
library(gridExtra)

#study design
#############
#number of measurement
K<-7
#points of time afterintervention
T.points<-K-1

#parameter Fidelity specification
###############################
Fid.T1<-0.2
Fid.End<-1

####set several slopes of increasing fidelity
#######################################
slope.seq<-round(exp(1)^(seq(-2,2,1)),2)
nr.sl<-length(slope.seq)
```

```
####fidelity patterns determined by several slopes within slow and fast increase
##############################################################################
res.plot.Patterns<-NULL
for(sl in slope.seq){
 res<-fidelitysim::find.Fidelity.log(time.points=T.points, Fid.End, Fid.T1,
                                     par.slope=sl)
 res<-data.frame(res, FUN="log", slope=sl)
 res.plot.Patterns<-rbind(res.plot.Patterns, res)
 res<-fidelitysim::find.Fidelity.exp(time.points=T.points, Fid.End, Fid.T1,
                                     par.slope=sl)
 res<-data.frame(res, FUN="exp", slope=sl)
 res.plot.Patterns<-rbind(res.plot.Patterns, res)

}

#fidelity pattern for linear increase
#####################################
res.lin<-fidelitysim::find.Fidelity.linear(time.points=T.points, Fid.End, Fid.T1)
res.plot.Patterns<-rbind(res.plot.Patterns, data.frame(res.lin, FUN="linear",
                                                       slope=1))
```

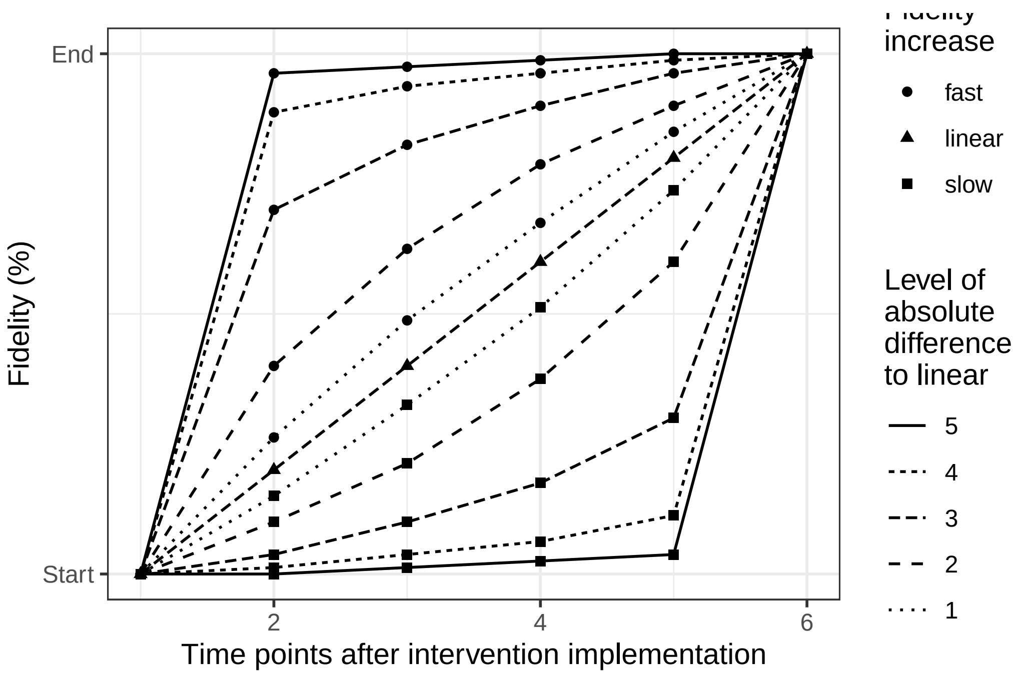

Figure 3.1: Patterns of a fidelity increase (fast, linear or slow) over 6 times points

For our calculation within the simulation we use the determined fractional values of intervention effects to define the degree of deviation from 100% implementation within the design matrix. Three functions in {fidelitysim} provide the implementation of the different patterns of fidelity.

3.3.1 Slow increase of fidelity using exponential function

Here we show an example how to determine the design matrix for a cluster randomized **parallel design** study (with same design as above) with existing implementation error, where fidelity starts with **40% after implementation** and reach after a slow increase **80% at the end** of the study. A great slope parameter is chosen which reflect a **slow increase more closed to the linear** increase.

```
#study design
#############
I <- 6 # number of clusters
J <- 10 # number of individuals per cluster (used later in simulation-step)
K <- 7 # number of time points
Sw<- round(I/2) # number of cluster within the control group

#parameter Fidelity specification
###############################
Fid.T1<-0.4
Fid.End<-0.8

#parameter tunes the slope for the log and exp functions
slope.seq<-5

#exponential function to determine slow increase
(res.exp<-fidelitysim::find.Fidelity.exp(time.points=K, Fid.End, Fid.T1,
                                         par.slope=slope.seq))
```

```
     time Fidelity.Prozent
[1,]    1               40
[2,]    2               44
[3,]    3               49
[4,]    4               55
[5,]    5               62
[6,]    6               70
[7,]    7               80
```

```
#determine correspondingdesign matrix
(A1.exp <-fidelitysim::implemMatrix.parallel(nC=I, nT=K, nSw=Sw,
                                pattern=res.exp[,"Fidelity.Prozent"]/100))
```

```
     [,1] [,2] [,3] [,4] [,5] [,6] [,7]
[1,]  0.0 0.00 0.00 0.00 0.00  0.0  0.0
[2,]  0.0 0.00 0.00 0.00 0.00  0.0  0.0
[3,]  0.0 0.00 0.00 0.00 0.00  0.0  0.0
[4,]  0.4 0.44 0.49 0.55 0.62  0.7  0.8
[5,]  0.4 0.44 0.49 0.55 0.62  0.7  0.8
[6,]  0.4 0.44 0.49 0.55 0.62  0.7  0.8
```

3.3.2 Linear increase of fidelity using linear function

Here we show an example how to determine the design matrix for a cluster randomized **parallel design** study (with same design as above) with existing implementation error, where fidelity starts with **40% after implementation** and reach after a **linear** increase **80% at the end** of the study.

```
#parameter Fidelity specification
################################
Fid.T1<-0.4
Fid.End<-0.8


#slope for linear function
m<-(Fid.T1-Fid.End)/(1-(K-1))

#linear increase
(res.lin<-fidelitysim::find.Fidelity.linear(time.points=K, Fid.End, Fid.T1))
```

```
     time Fidelity.Prozent
[1,]    1               40
[2,]    2               47
[3,]    3               53
[4,]    4               60
[5,]    5               67
[6,]    6               73
[7,]    7               80
```

```
# design matrix of a learning impelementation pattern, linear
(A1.lin <-fidelitysim::implemMatrix.parallel(nC=I, nT=K, nSw=round(I/2),
                                    pattern=res.lin[,"Fidelity.Prozent"]/100))
```

```
     [,1] [,2] [,3] [,4] [,5] [,6] [,7]
[1,]  0.0 0.00 0.00  0.0 0.00 0.00  0.0
[2,]  0.0 0.00 0.00  0.0 0.00 0.00  0.0
[3,]  0.0 0.00 0.00  0.0 0.00 0.00  0.0
[4,]  0.4 0.47 0.53  0.6 0.67 0.73  0.8
[5,]  0.4 0.47 0.53  0.6 0.67 0.73  0.8
[6,]  0.4 0.47 0.53  0.6 0.67 0.73  0.8
```

3.3.3 Fast increase of fidelity using logarithmic function

Here we show an example how to determine the design matrix for a cluster randomized **stepped wedge design** study (with same design as above) with existing implementation error, where fidelity starts with **20% after implementation** and reach after a fast increase **100% at the end** of the study. A small slope parameter is chosen for determining the fidelity curve, which reflect a **very fast increase**.

```
Sw <- 1 # number of cluster switches per time point can be manually set

#parameter Fidelity specification
###############################
Fid.T1<-0.2
Fid.End<-1

#parameter tunes the slope for the log and exp functions
slope.seq<-0.2

#logistic function to determine fast increase
(res.log<-fidelitysim::find.Fidelity.log(time.points=K-1, Fid.End, Fid.T1,
                                         par.slope=slope.seq))
```

```
     time Fidelity.Prozent
[1,]    1               20
[2,]    2               95
[3,]    3               97
[4,]    4               98
[5,]    5               99
[6,]    6              100
```

```r
(A1.log <-samplingDataCRT::implemMatrix.SWD(nC=I, nT=K, nSw=Sw,
                                            pattern=res.log[,"Fidelity.Prozent"]/100))
```

```
     [,1] [,2] [,3] [,4] [,5] [,6] [,7]
[1,]    0  0.2 0.95 0.97 0.98 0.99 1.00
[2,]    0  0.0 0.20 0.95 0.97 0.98 0.99
[3,]    0  0.0 0.00 0.20 0.95 0.97 0.98
[4,]    0  0.0 0.00 0.00 0.20 0.95 0.97
[5,]    0  0.0 0.00 0.00 0.00 0.20 0.95
[6,]    0  0.0 0.00 0.00 0.00 0.00 0.20
```

References

Bates, Douglas, Martin Mächler, Ben Bolker, and Steve Walker. 2015. "Fitting Linear Mixed-Effects Models Using lme4." *Journal of Statistical Software* 67 (1): 1–48. https://doi.org/10.18637/jss.v067.i01.

Hussey, Michael A., and James P. Hughes. 2007. "Design and Analysis of Stepped Wedge Cluster Randomized Trials." *Contemporary Clinical Trials* 28 (2): 182–91. https://doi.org/https://doi.org/10.1016/j.cct.2006.05.007.

R Core Team. 2022. *R: A Language and Environment for Statistical Computing*. Vienna, Austria: R Foundation for Statistical Computing. https://www.R-project.org/.

Trutschel, Diana, and Catherine Blatter. 2022. *fidelitysim: Simulations Experiments for Cluster Randomized Trials Having Fidelity Patterns for Implementation Science Research*.

Trutschel, Diana, and Hendrik Treutler. 2017. *samplingDataCRT: Sampling Data Within Different Study Designs for Cluster Randomized Trials*. https://CRAN.R-project.org/package=samplingDataCRT.

Wickham, Hadley. 2016. *Ggplot2: Elegant Graphics for Data Analysis*. Springer-Verlag New York. https://ggplot2.tidyverse.org.
