# Supplementary material for: The unrecognized role of fidelity in effectiveness-implementation hybrid trials: simulation study and guidance for implementation researchers
Source: BMC Med Res Methodol. 2023 May 13;23:116. doi: 10.1186/s12874-023-01943-3 (PMC10183132; doi:10.1186/s12874-023-01943-3)
Supplement: Supplementary file 2 — Supplementary Material 2 [file 12874_2023_1943_MOESM2_ESM.pdf]

Additional File 1

Supplementary method description

Simulation study

The simulation study described in the methods section explores two study designs, i.e. parallel and stepped-wedge cluster randomized trials, and hypothetical fidelity patterns (slow, linear, fast) using fixed design parameters, i.e., the number of clusters (C=6), time points (T=7) and patients per cluster (n=10). Figure S1 provides an overview of the overall workflow of the simulation study.

The provided online tutorial explains the application of the simulation to get results for one specific fidelity pattern (Section 2.1 in online tutorial) as well as the comparison of several fidelity patterns (Section 2.2 in online tutorial) for one specific design. In the article several designs are included and compared.

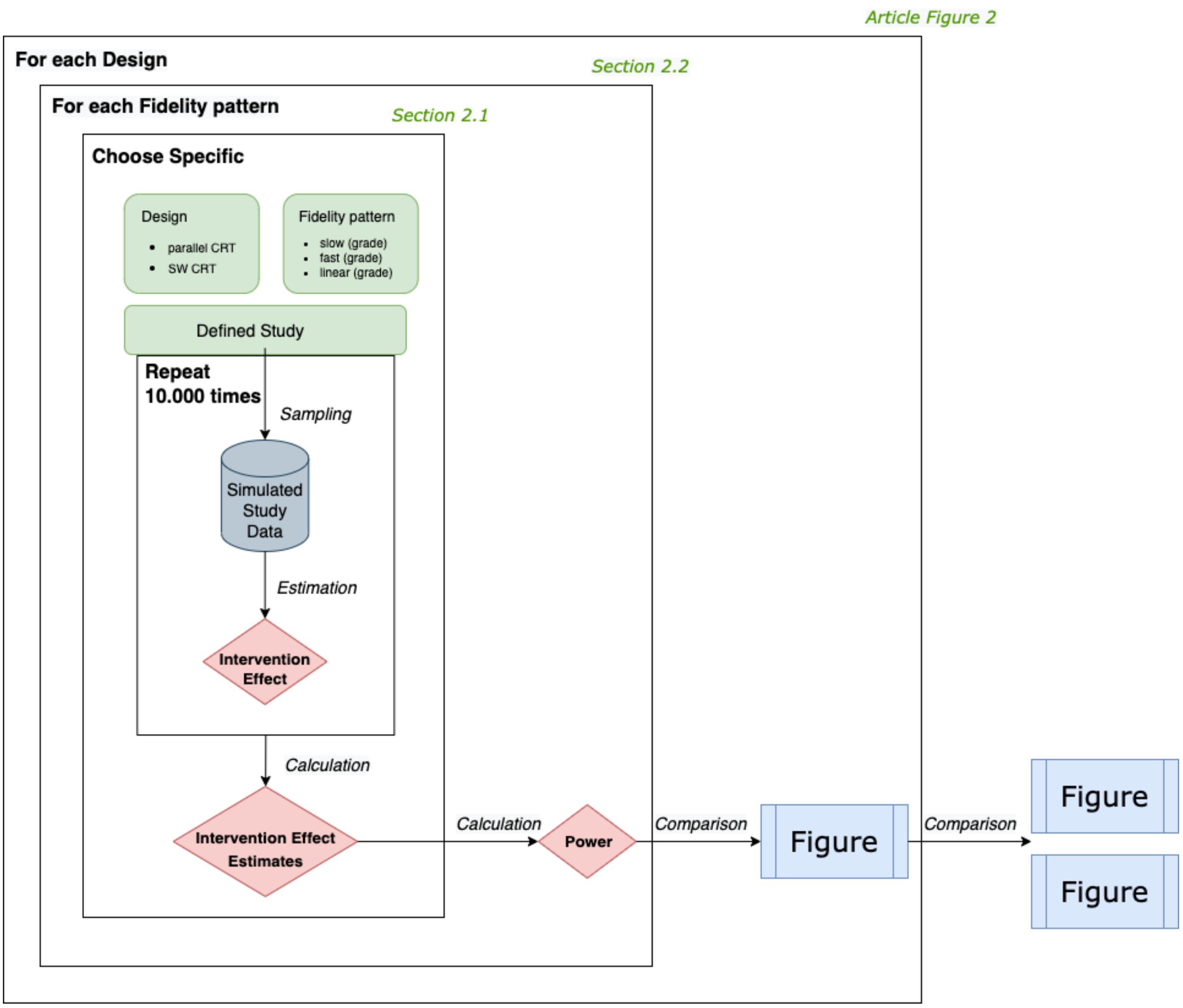

Figure S1: Workflow of the simulation study

Functions for patterns of fidelity over time

We used an exponential, linear and logarithmic function with different slopes to model slow, linear and fast fidelity increase (Figure S2). The graph of a linear function is a straight line with a slope of 1. The logarithmic function is, in general, the inverse function of the exponential function and can be obtained by mirroring the points of the exponential function on the linear function as the mirror axis (Figure S2 left). We used several shifts of the derived functions to determine the final fidelity patterns for given start and end y-values (Figure S2 right).

For all three functions, we can determine the fidelity at each time point x_i with mathematical derivation $y = f(x_i)$, given the number of time points, the fidelity at the starting point (fidelity assessed at first time point after intervention was implemented) and the end point (fidelity assessed at the last time point), explained in the following.

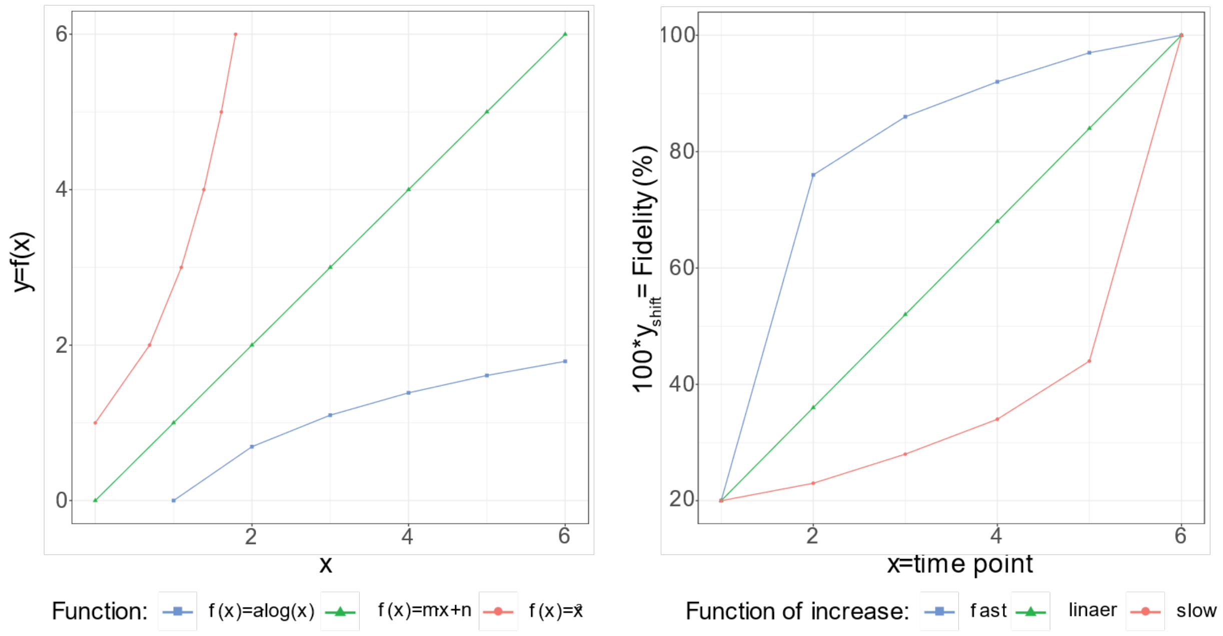

Figure S2: Exemplary illustration of an exponential function $f(x) = x^a$, linear function $f(x) = m \cdot x + n$ and logarithmic function $f(x) = a \cdot log(x)$ (left figure); The same functions after shifting represent different fidelity patterns for slow, linear and fast increase (right figure).

Linear function

The slope parameter m and the intercept n of a linear function

$$y = f(x) = m \cdot x + n$$

can be determined by two points $P_1(x_1, y_1)$ and $P_2(x_2, y_2)$ of the function given as follows

$$m = \frac{y_2 - y_1}{x_2 - x_1} \text{ and } n = y_1 - m \cdot x_1.$$

For any given example of fidelity, $fidelity_{start}$ and $fidelity_{end}$, we define two points of the function using the starting time and end time point of the study as x-values and their corresponding fidelity as y-values:

$$P_1(x_1 = 1, y_1 = fidelity_{start}) \text{ and } P_2(x_2 = T, y_2 = fidelity_{end}),$$

where T is number of time points of the study after introduction of intervention. Finally, given all time points $x = 1, \cdots, T$, we can determine the fidelity value at each time point x_i from the derived function $f(x)$.

Logarithmic function

A logarithmic function

$$y = f(x) = a \cdot \log(x)$$

is used to model a fast fidelity increase for the time points $x = 1, \cdots, T$. Here the slope parameter a determines the shape of the logarithmic function and thus the fidelity pattern.

Furthermore, scaling and shifting the derived functional values y by

$$y_{shift} = c \cdot y + b,$$

where b is the fidelity at the start measurement point $fidelity_{start}$ and c a normalisation parameter determined by $c = \frac{fidelity_{end} - fidelity_{start}}{f(T) - f(1)}$. Both ensures that the fidelity values are between the start and end measurement $(fidelity_{start}, fidelity_{end})$.

Exponential function

As stated above, the exponential and the logarithmic function have common characteristics Therefore, to model a hypothetical increase of fidelity, values are determined with the corresponding logarithmic function as a basis. For this, the order of the function values $y_i = f_{linear}(x_i)$ are reversed and shifted in direction of the y-axis by adding the difference of the logarithm function value to the linear function value $f_{linear}(x_i) - f_{logarithm}(x_i)$ to each linear function value $y_i = f_{linear}(x_i)$ (Figure S2).
